# Transmissible Gastroenteritis Virus Papain-Like Protease 1 Antagonizes Production of Interferon-*β* through Its Deubiquitinase Activity

**DOI:** 10.1155/2017/7089091

**Published:** 2017-10-23

**Authors:** Xiaoliang Hu, Jin Tian, Hongtao Kang, Dongchun Guo, Jiasen Liu, Dafei Liu, Qian Jiang, Zhijie Li, Juanjuan Qu, Liandong Qu

**Affiliations:** ^1^State Key Laboratory of Veterinary Biotechnology, Harbin Veterinary Research Institute, Chinese Academy of Agricultural Sciences, Harbin 150001, China; ^2^College of Life Science, Northeast Agricultural University, Harbin, China

## Abstract

Coronaviruses (CoVs), such as human coronavirus NL63 (HCoV-NL63), severe acute respiratory syndrome CoV (SARS-CoV), murine hepatitis virus (MHV), porcine epidemic diarrhea virus (PEDV), and Middle East Respiratory Syndrome Coronavirus (MERS-CoV), encode papain-like (PL) proteases that inhibit Sendai virus- (SeV-) induced interferon (IFN-*β*) production. Recently, the crystal structure of transmissible gastroenteritis virus (TGEV) PL1 has been solved, which was similar to that of SARS-CoV PL2^pro^, which may antagonize host innate immunity. However, very little is known about whether TGEV PL1 can antagonize host innate immune response. Here, we presented evidence that TGEV PL1 encoded by the replicase gene could suppress the IFN-*β* expression and inhibit the nuclear translocation of interferon regulatory factor 3 (IRF3). The ability to antagonize IFN-*β* production was dependent on the intact catalytic activity of PL1. Furthermore, TGEV PL1 exerted deubiquitinase (DUB) activity which strongly inhibited the retinoic acid-induced gene I- (RIG-1-) and stimulator of interferon gene- (STING-) dependent IFN expression. Our data collectively suggest that TGEV PL1 can inhibit the IFN-*β* expression and interfere with RIG-1- and STING-mediated signaling through a viral DUB activity. Our study has yielded strong evidence for the TGEV PL1 mechanisms that counteract the host innate immunity.

## 1. Introduction

The innate immune system is the first line of defense that protects the host against viral infection, and the induction of IFN-*α*/*β* is a crucial antiviral mechanism of the innate immune system [1]. The initiation of IFN expression is triggered by pathogen-associated molecular patterns (PAMPs) through host pattern recognition receptors (PRRs) [2]. After viral RNAs are sensed by PRRs, signals are transmitted to different downstream adaptor molecules (such as IFN-*β* promoter stimulator 1 (IPS-1)); and then I*κ*B kinase- (IKK-) related kinases are recruited. Next, interferon regulatory factor 3 (IRF3), nuclear factor *κ*B (NF-*κ*B), and ATF-2/c-jun are activated by the kinase complexes and translocate to the nucleus and directly induce the expression of type I IFNs [3].

TGEV is an enveloped virus belonging to the Coronaviridae (CoV) family and the Nidovirales order. CoVs are positive-strand RNA viruses that replicate in the cytoplasm of infected cells [4]. CoVs encode two types of cysteine proteases, M^pro^, and papain-like proteases, PL1 and PL2, which contained nonstructural protein 5 (nsp5) and nsp3, respectively. PL^pro^ is served mainly as in processing of the replicase pp1a and pp1ab polypeptides [5]. Other than their role in replicase polyprotein processing, PL2 domains possess an additional but related enzymatic activity, in HCoV-NL63 [6], MHV [7], SARS-CoV [8, 9], and MERS-CoV [10], through their deubiquitination (DUB) enzymes, which play a key role in antagonizing IFN induction. However, TGEV PL1 processes the nsp2/nsp3 site and is capable of hydrolyzing isopeptide bonds in both Lys48- and Lys63-linked polyubiquitin chains [11]. Whether TGEV PL1 could antagonize the production of IFNs was unknown.

In the present study, we found that TGEV PL1 encoded by the replicase gene could suppress the IFN-*β* expression and inhibit the nuclear translocation of interferon regulatory factor 3 (IRF3) and exerted deubiquitinase (DUB) activity which strongly inhibited the retinoic acid-induced gene I- (RIG-1-) and stimulator of interferon gene- (STING-) dependent IFN expression.

## 2. Methods

*Cells and Viruses.* HEK293T cells and PK-15 cells were cultured in Dulbecco's modified Eagle's medium (Hyclone, Logan, USA) containing 10% (v/v) fetal calf serum supplemented with penicillin (100 U ml^−1^) and streptomycin (100 *μ*g ml^−1^). Sendai virus (SeV) was obtained from the Centre of Virus Resource and Information (Wuhan Institute of Virology, Chinese Academy of Sciences).

*Plasmids and Agents*. IFN-*β*-Luc, 4x PRDIII/I-Luc (referred to as IRF3-Luc), 4x AP-1-Luc, and 4x NF-*κ*B-Luc luciferase reporter plasmids were constructed according to an earlier protocol [12]. Accession numbers of STING, IRF3, and MAVS were KC860780, KC860781, and KC860782, respectively. Expression plasmids for RIG-1 (p-Flag-RIG-1) and TBK-1 (p-Flag-TBK-1) were generated with the following primers: RIG-1 forward, 5′-TTTGGATCCATGACAGCAGAGCAGCGGCGGAAT-3′, RIG-1 reverse 5′-TTTAAGCTTCACTCAAGGTTCGGGATTCCCTG-3′; TBK-1 forward, 5′-TTTGAATTCATGCAGAGCACTTCTAATCATCTTT-3′, TBK-1 reverse 5′-TTTAGATCTTAAAGACAGTCAACATTGCGAA-3′. To construct the DNA expression vector, pMyc-PL1, pFlag-PL1, and pPL1-Myc, encoding TGEV PL1, standard reverse transcription- (RT-) PCR was applied to amplify cDNA of the total RNA extracted from PK-15 cells infected with the TGEV strain HX, using the following primers: PL1-forward, 5′-GTACAAGAAGCTGAACAATTTAA-3′ (3498–3520 bp), PL1 reverse, 5′-ATCGTTTTTAGGACTTTGAATTT-3′ (4249–4271 bp). All constructs were validated via DNA sequencing. pDsRed2-Mito was purchased from Clontech (Tokyo, Japan). Transfection agent was performed with X-tremeGENE HP (Roche, Switzerland) per the manufacturer's instructions.

*Luciferase Reporter Gene Assay*. HEK-293T cells grown in 24-well plates were cotransfected with 0.2 *μ*g/well reporter plasmid, 0.02 *μ*g/well pRL-TK plasmid (Promega, Madison, USA) as an internal control for normalization of transfection efficiency, and the indicated expression or empty control vector plasmid. Where indicated, cells were also mock-infected or infected with SeV (100 hemagglutinating activity units/well) at 10 h after cotransfection. Cells were subsequently lysed, and firefly and* Renilla* luciferase activities were determined with the Dual-Luciferase reporter assay system (Promega, Madison, USA), according to the manufacturer's protocol. Data are presented as mean relative luciferase units ± standard deviation from triplicate samples. For statistical analysis, data were compared between empty vector- and TGEV PL1-transfected groups with the unpaired, two-tailed Student's* t*-test using SPSS 11.0 software. *P* values < 0.05 were considered statistically significant [13].

*ELISA*. Cell supernatants of transfected PK-15 cells were centrifuged at 3,000*g* for 5 min to remove cell debris and stored at −80°C until use. Secreted IFN-*β* in the cell supernatants was determined using commercial Porcine IFN-*β* (Interferon Beta) ELISA Kit (Elabscience, China) according to the manufacturer's instructions.

*Immunoblot Analysis*. HEK293T cells were cultured in 6-well plates and 60 mm dishes were transfected with the appropriate plasmids. After 36 h, cells were harvested by the addition of lysis buffer and protein concentrations in whole cell extracts measured. Equal amounts of samples were subjected to SDS-PAGE and analyzed for TGEV PL1, STING, TBK-1, and IRF3 proteins via immunoblotting using HA, Flag, or GFP-tagged antibodies (Sigma, St Louis, USA). Expression of p-IRF3, IRF3, and GAPDH was detected with the rabbit-anti p-IRF3 (ab76493), IRF3 (ab68481) (Abcam, Cambridge, UK), and a mouse anti-GAPDH monoclonal antibody (Sigma, St Louis, USA).

*Assay of Deubiquitinase Activity in Cultured Cells*. HEK293T cells were cotransfected with pcDNA3.1-HA-Ub plus the indicated amounts of TGEV PL1, p-Flag-RIG-1, and p-Flag-STING constructs. The effect of TGEV PL1 on ubiquitinated proteins in cultured cells was assessed by immunoblot analysis.

*Coimmunoprecipitation Analysis*. Coimmunoprecipitation experiments were performed on HEK293T cells transfected with the indicated expression plasmids as described in an earlier report [14].

*Immunofluorescence Assay*. HEK293T cells were plated on fibronectin-treated glass coverslips in 24-well plates. To evaluate the localization of TGEV PL1, cells were cotransfected with plasmid DNA expressing Flag-PL (500 ng per well) and pDsRed-Mito (500 ng per well) using X-treme GENE HP, according to the manufacturer's protocol. HEK293T cells were cotransfected with IRF3-GFP (500 ng per well) and empty vector (500 ng per well) or IRF3-GFP (500 ng per well) and Flag-PL1 (500 ng per well). 24 h after transfection, cells were infected with SeV (100 hemagglutinating activity units/well) for 16 h. Next, cells were fixed with 4% paraformaldehyde for 30 min and permeated with 0.1% Triton X-100 for 15 min at room temperature. After three washes with PBS, cells were blocked with PBS containing 5% bovine serum albumin for 2 h, followed by incubation with a mouse monoclonal antibody against Flag (1 : 100) for 1 h at room temperature. Cells were treated with fluorescein isothiocyanate-labeled goat anti-mouse (Sigma, St Louis, USA) for 1 h, and subsequently with 4′,6-diamidino-2-phenylindole (DAPI) for 15 min at room temperature. Samples were washed with PBS, and fluorescent images were acquired under a confocal laser scanning microscope (TCS SP5; Leica, Solms, Germany).

*Detection of STING Dimers*. To assess the formation of STING dimers, HEK293T cells were transfected with Flag-STING (500 ng per well) and the lysates were subjected to Western blot, as described earlier [15], with the indicated antibodies.

## 3. Results

### 3.1. TGEV PL1 Is an IFN Antagonist

**Figure 1:**
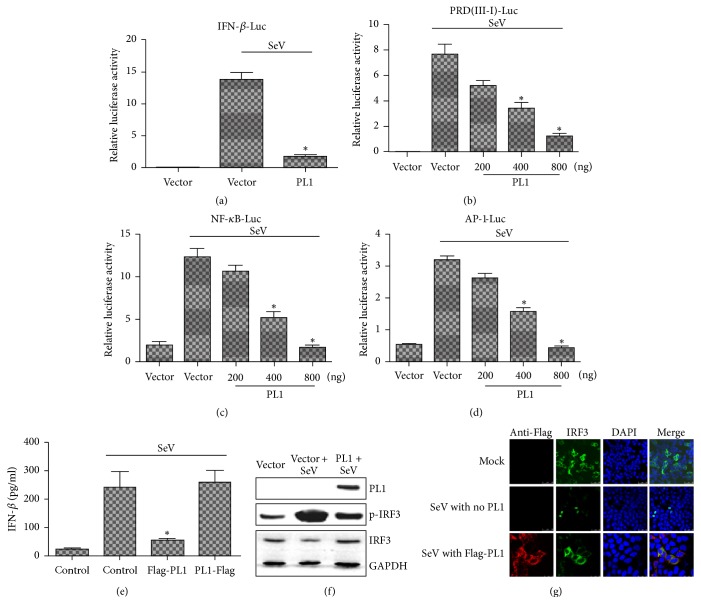
*TGEV PL1 inhibits SeV-induced expression of IFN-β-Luc in a dose-dependent manner.* (a) HEK-293T cells grown in 24-well plates were transfected with 1 *μ*g plasmid encoding Myc-TGEV PL1 or empty vector and infected with SeV 24 h later (100 hemagglutinating activity units/well). After 10 h infection, cells were lysed, and activities of IFN-*β*-Luc and pRL-TK reporters were determined according to the manufacturer's protocol. Myc-TGEV PL1 inhibits the activities of IRF3 (b), NF-*κ*B (c), and AP-1 (d). Luciferase activities were assayed as described for (a). Results represent the means and standard deviations of data from three independent experiments. (e) PK-15 cells grown in 12-well plates were transfected with 500 ng plasmid Flag-PL1 and empty vector and infected with SeV 24 h later. After 10 h infection, cell supernatants were collected and analyzed for IFN-*β* production by ELISA. (f) HEK-293T cells grown in 6-well plates were transfected with 2 *μ*g plasmid encoding Myc-TGEV PL1 or empty vector and infected with SeV 24 h later (100 hemagglutinating activity units/well). After 10 h infection, cells were lysated and detected by Western blot. (g) Immunofluorescence microscopy of HeLa cells expressing Flag-PL1 and IRF3-GFP. Cells were fixed 24 h after transfection and 10 h SeV infection, and Flag-tagged products were visualized using confocal microscopy. Asterisks indicate statistical significance (*P* < 0.05).

The crystal structure of TGEV PL1 has been determined [11]. The structure of TGEV PL1 is similar to that of SARS-CoV PL2^pro^. In order to determine whether TGEV PL1 is capable of blocking IFN-*β* production, we assessed IFN-*β* promoter activity in the presence of PL1 (Figure 1(a)). HEK 293T cells were cotransfected with TGEV PL1 and IFN-*β* luciferase or Renilla luciferase reporter plasmids for 24 h and subsequently infected with SeV to activate the RIG-1-dependent IFN-*β* expression pathway. We observed the inhibition of SeV-induced IFN-*β* promoter activation in the presence of PL1, similar to the antagonistic function of NL63 PLP2 and porcine epidemic diarrhea virus (PEDV) PLP2, clearly indicating that TGEV PL1 could act as an interferon antagonist.

To establish the mechanisms by which PL1 inhibits IFN-*β* expression, transcriptional activities of NF-*κ*B, IRF3, and AP-1 were analyzed using the luciferase assay to identify the precise transcription factor involved. Notably, the luciferase activities of all three transcription factors were significantly inhibited by TGEV PL1 in a dose-dependent manner (Figures 1(b), 1(c), and 1(d)). Furthermore, Flag-PL1 also significantly inhibited IFN-*β* production in PK-15 cells at protein level (Figure 1(e)), which was further confirmed with the result that TGEV PL1 could block the production of interferon. To further establish whether TGEV PL1 affects IRF3 phosphorylation or migration from the cytoplasm to nucleus, HEK293T cells were transfected with TGEV PL1 and/or IRF3-EGFP. Then the result was analyzed using Western blot and confocal microscopy. In Figure 1(f), the level of p-IRF3 was decreased significantly by TGEV PL1 compared with that of SeV-induced. Furthermore, IRF3-EGFP was located in the cytoplasm compared with mock-infected HEK293T cells but translocated to the nucleus when the cells were inoculated with SeV. In contrast, after being inoculated with SeV, it was found that IRF3-EGFP was translocated from cytoplasm to nuclear in mock infected HEK293T cell, which was not observed in cells transfected with TGEV PL1 (Figure 1(g)). Our results collectively suggested that TGEV PL1 suppressed IFN-*β* transcription by interfering with NF-*κ*B-, IRF3-, and AP-1 signaling-mediated IFN expression.

### 3.2. TGEV PL1 Antagonized STING-Dependent Signaling

**Figure 2:**
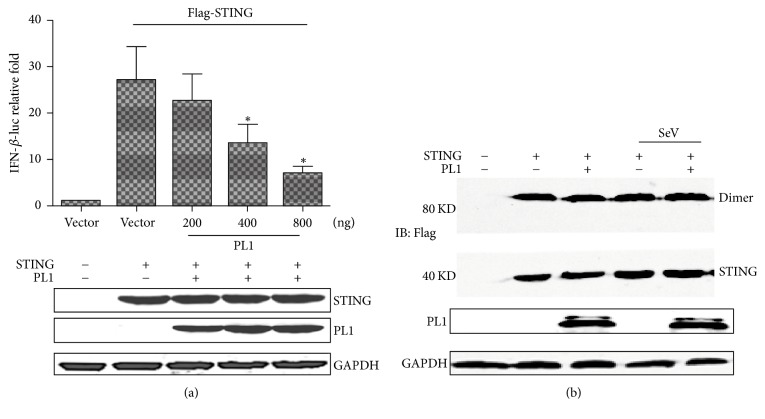
*Expression of TGEV PL1 inhibits STING-mediated activation of IFN-β-Luc in a dose-dependent manner.* (a) HEK293T cells were cotransfected with certain Flag-PL1, IFN-*β*-Luc, pRL-TK, and 500 ng Flag-STING or 500 ng empty vector. Asterisks indicate statistical significance (*P* < 0.05). Proteins were assayed using Western blot with anti-Flag and GAPDH antibodies. (b) HEK293T cells were cotransfected with 500 ng STING and/or 500 ng TGEV PL1, and/or infected with SeV. Cell lysates were separated via SDS-PAGE and subjected to immunoblotting with the relevant antibodies.

To determine whether TGEV PL1 is capable of blocking STING-mediated activation of the IFN-*β* promoter, we assessed promoter activity in the presence of STING along with increasing amounts of TGEV PL1. Stimulation of HEK293T cells with STING alone resulted in a robust increase in IFN-*β* promoter activity. Coexpression of STING and TGEV PL1 induced a dose-dependent decrease in IFN-*β* activity, clearly indicating antagonistic activity of TGEV PL1 on STING-mediated activation of the IFN-*β* promoter (Figure 2(a)).

STING dimerization is reduced in the presence of HCoV-NL63. STING dimmers are visualized as an 80 kD band on SDS-PAGE. To further determine whether TGEV PL1 inhibits STING-mediated signaling through disrupting the stability of STING dimers, HEK293T cells were cotransfected with plasmid DNA expressing STING in the presence or absence of TGEV PL1 and SeV, and cell lysates were evaluated for dimmers via immunoblotting (Figure 2(b)). Interestingly, the results indicated that STING dimerization was not affected by TGEV PL1.

### 3.3. The Catalytic Activity of TGEV PL1 Is Essential for Inhibiting IFN-*β* Expression

**Figure 3:**
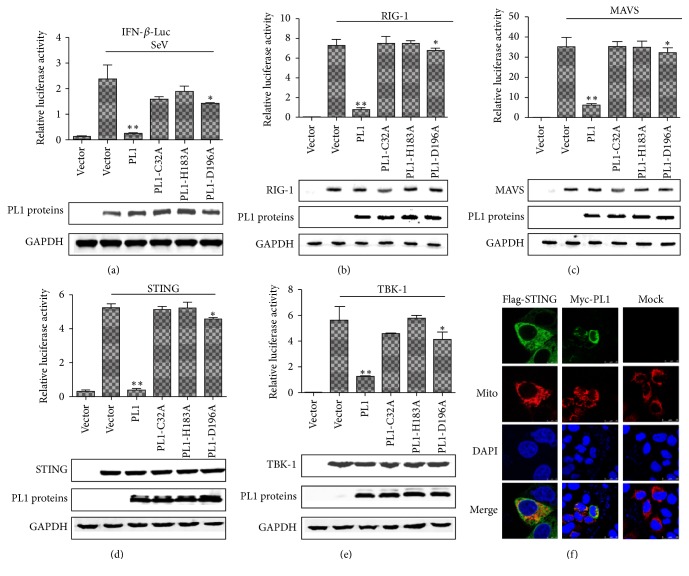
*Effects of the TGEV PL1 catalytic mutants on expression of the IFN-β-Luc and localization of the protein.* (a) HEK293T cells were cotransfected with the 500 ng catalytic mutants C32A, H183A, and D196A, together with reporters of IFN-*β*-Luc and pRL-TK. Asterisks indicate statistical significance (*P* < 0.05). (b, c, d, e) HEK293T cells were transfected separately with RIG-1 (500 ng), MAVS (500 ng), STING (500 ng), or TBK-1 (500 ng), together with IFN-*β*-Luc and pRL-TK. Asterisks indicate statistical significance (*P* < 0.05). (f) Immunofluorescence microscopy of HeLa cells expressing Myc-TGEV PL1, Flag-STING, and DsRed-Mito. Cells were fixed 24 h after transfection, and Flag-tagged and Myc-tagged products were visualized using confocal microscopy.

To determine whether catalytic activity is required for TGEV PL1-mediated inhibition of IFN-*β* expression, HEK293T cells were cotransfected with alanine mutants of three conserved catalytic residues of TGEV PL1 (C32A, H183A, and D196A) with or without RIG-1, MAVS, STING, or TBK-1, and IFN-*β*-Luc and pRL-TK plasmids, followed by infection with SeV to activate IFN-*β* promoter activity. TGEV PL1 mutation at two of the catalytic sites (C32A and H183A) led to almost complete loss of IFN antagonistic activity, relative to wild-type TGEV PL1, but the D196A mutant showed a little inhibition for IFN-*β* promoter activity (Figures 3(a), 3(b), 3(c), 3(d), and 3(e)). Based on the results, we conclude that the intact catalytic triad of TGEV PL1 is required to inhibit activation of the IFN-*β* promoter driven by STING and TBK-1. Recent studies have revealed that STING acts as a scaffold protein for TBK-1 and IRF3 and links them to the MAVS complex in mitochondria upon viral infection [16]. Moreover, activation of STING is critical for stimulation of IRF-3 activity. Here, we observed that TGEV PL1 protein inhibits STING- and TBK-1- induced activation of IFN-*β*. Additional localization experiments showed that PL1 existed in mitochondria (Figure 3(f)).

### 3.4. TGEV PL1 Binds and Deubiquitinates RIG-I and STING

**Figure 4:**
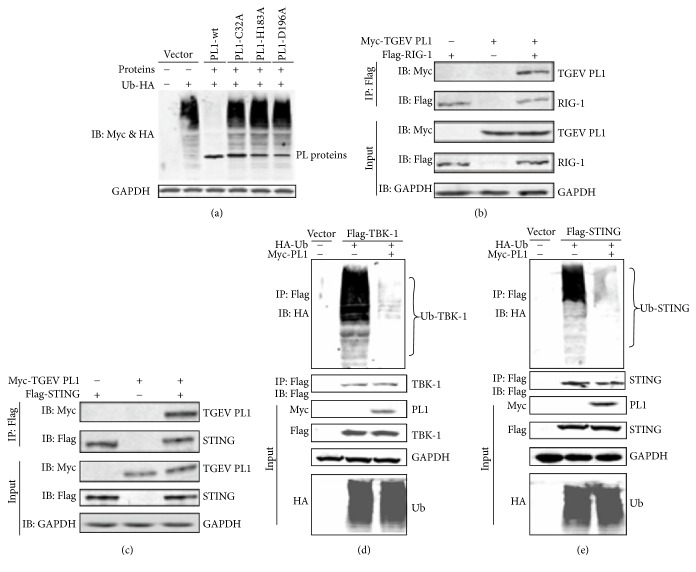
*TGEV PL1 displays DUB activity that is dependent on its catalytic activity and reduces the ubiquitinated forms of RIG-I and STING.* (a) HEK293T cells were transfected with HA-tagged ubiquitin and TGEV PL1 or the catalytic mutants C32A, H183A, and D196A. Proteins were assayed using Western blot with anti-HA and anti-Myc antibodies. ((b) and (c)) HEK293T cells were transfected with Myc-TGEVPL1 together with Flag-RIG-1 (b) and Flag-STING (c) for 24 h, and lysates were subjected to coimmunoprecipitation and Western blot to determine the ubiquitination status of immunoprecipitated proteins. ((d) and (e)) HEK293T cells were transfected with Flag-RIG-1 (d) or Flag-STING (e), together with HA-Ub in the presence or absence of Myc-TGEVPL1. Cells were incubated for 24 h after transfection and then lysates were harvested. Lysates were immunoprecipitated with Flag, HA, and Myc antibodies and the products subjected to immunoblotting with anti-HA to evaluate ubiquitinated proteins.

Modification of signaling molecules by ubiquitin (Ub) plays a critical role in activation of the IFN response. TGEV PL1 has been shown to possess DUB activity. Here, we investigated the DUB activities of TGEV PL1 and its catalytic mutants. HEK293T cells were cotransfected with pcDNA HA-Ub and TGEV PL1, and the level of ubiquitinated proteins was assessed via Western blot. The level of Ub-conjugated proteins was reduced dramatically in cells transfected with wild-type TGEV PL1, while the ubiquitinated Ub-HA level was not reduced in the presence of the C32A, H183A, and D196A mutants (Figure 4(a)). Next, we investigated whether TGEV PL1 recognizes and deubiquitinates the key regulators, RIG-I and STING, in the IFN signaling pathway. HEK293T cells were transfected with TGEV PL1, together with Flag-RIG-1 and Flag-STING for 24 h, and cell lysates were subjected to coimmunoprecipitation and Western blot to determine the ubiquitination of the immunoprecipitated protein. TGEV PL1 was detected in association with RIG-1 as well as STING (Figures 4(b) and 4(c), lane 3). Moreover, a dramatic reduction in the amount of ubiquitinated RIG-1 (Figure 4(d)) and STING (Figure 4(e)) was detected. These results suggest that TGEV PL1 antagonizes the IFN signaling pathway via deubiquitination of RIG-1 and STING.

## 4. Discussion

TGEV is known to induce robust expression of IFN-*α* at the late step of the replication and is distinct from CoVs [17, 18]. Moreover, TGEV infection activates transcription factors NF-*κ*B, IRF3, and AP-1 in porcine kidney cells and a delayed activation of the IFN response in intestinal epithelial cells [19, 20]. However, the mechanism of its evasion of the innate immune system has never been reported. The current study firstly showed antagonistic function of the TGEV PL1 protein against the IRF3 signaling pathway to inhibit IFN-*β* induction through its DUB activity.

To combat the host antiviral effects, coronaviruses likely take advantage of PL activity to escape from the host innate antiviral response. HCoV-NL63 (PL2-TM) and SARS-CoV (PLpro-TM) inhibit STING-mediated activation of IRF-3 nuclear translocation and induction of IRF-3-dependent promoters [6, 8]. PL2 of MHV strongly inhibits CARDIF-, TBK1-, and IRF3-mediated IFN-*β* reporter activities and prevented nuclear translocation of IRF3 [7]. PEDV PLP2 negatively regulated RIG-I and STING-mediated IFN-*β* expression [14]. Moreover, TGEV PL1 displays a similar structure to SARS-CoV PL2 [11] and gives rise to the speculation that TGEV PL1 may similarly act as an IFN antagonist. In the present study, we first found that overexpressed TGEV PL1 inhibited STING- and TBK-1-mediated IFN-*β* transcription and antagonized the type I IFN response stimulated by SeV in PK-15 cells. The catalytic activity of TGEV PL1 is essential for inhibiting IFN-*β* transcription. Furthermore, STING dimerization is reduced in the presence of HCoV-NL63 PL2-TM, which was not affected by TGEV PL1. These results suggested that TGEV PL1 acted as an IFN antagonist to negatively regulate host antiviral innate immunity.

Ubiquitination and deubiquitination are critically involved in regulation of virus-induced type I IFN signaling pathways [21, 22]. Recently, DUBs have been reported in a variety of viruses, such as foot-and-mouth disease virus, Lpro [23], human cytomegalovirus, UL48 [24], herpes simplex virus type 1, UL36 [25], and porcine reproductive and respiratory syndrome virus, nsp2 [26, 27]. Interestingly, all CoVs have evolved to encode DUB enzymes, which may contribute to modulation of the innate immune response. PLP of HCoV-NL63, SARS-CoV, MHV, PEDV, and MERS-CoV dramatically reduced the levels of ubiquitinated STING, RIG-I, TBK1, and IRF-3 to negatively regulate host antiviral innate immunity. Here, we showed that TGEV PL1 interferes with and significantly inhibits ubiquitination of RIG-1 and STING, which are essential activators of type I IFN signaling. Then, the levels of phosphorylated IRF-3 were reduced, which blocked nuclear translocation of IRF3 to activate the transcript of IFNs. Three catalytically inactive mutants of TGEV PL1 (C32A, H183A, and D196A) found to be defective in DUB activity failed to inhibit virus-induced INF-*β* expression, indicating that the DUB function of TGEV PL1 is directly involved in inhibition of type I IFN induction. However, the membrane protein M and envelope protein E of TGEV were translated at the late step of the replication as the major inducing component of IFNs. Further studies are required to establish the precise functions of PL1 protease/DUB activity in coronavirus interactions with the host innate immune response.

## 5. Conclusion

Our results are the first report identifying TGEV PL1 that is responsible for inhibiting the induction of IFN-*β*. We found that TGEV PL1 displayed IFN antagonist activity dependent on the intact catalytic triad (C32, H183, and D196) and interfered with RIG-1- and STING-mediated signaling through a viral DUB activity. These characteristics of TGEV PL1 served as a multifunctional protein with a critical regulatory role in TGEV interactions with the host antiviral innate immune response. Moreover, these findings contribute to our understanding of the molecular mechanisms of innate immunity evasion strategies utilized by TGEV.

## Abbreviations

CoVs: Coronaviruses
HCoV-NL63: Human coronavirus NL63
SARS-CoV: Severe acute respiratory syndrome CoV
MHV: Murine hepatitis virus
PEDV: Porcine epidemic diarrhea virus
MERS-CoV: Middle East Respiratory Syndrome Coronavirus
PL: Papain-like
SeV: Sendai virus
IFN: Interferon
TGEV: Transmissible gastroenteritis virus
IRF3: Interferon regulatory factor 3
DUB: Deubiquitinase
RIG-1: Retinoic acid-induced gene I
STING: Stimulator of interferon gene
PRR: Pattern recognition receptors
PAMP: Pathogen-associated molecular patterns
MDA5: Melanoma differentiation gene 5
IPS-1: IFN-*β* promoter stimulator 1
IKK: I*κ*B kinase
TBK1: TANK binding kinase 1
NF-*κ*B: Nuclear factor *κ*B
nsp5: Nonstructural protein 5.
